# Fatigue Induced by Walking Uphill and Downhill Similarly Disrupts Postural Balance but Their Disruptive Factors Differ

**DOI:** 10.3390/brainsci16080857

**Published:** 2026-08-13

**Authors:** Thierry Paillard, Aurélien Speller, Alex Rizzato, Giuseppe Marcolin, Julien Maitre

**Affiliations:** 1 Laboratoire Mouvement, Equilibre, Performance et Santé (UR 4445), Université de Pau & Pays de l’Adour, 11 rue Morane Saulnier, 65000 Tarbes, France; aurelien.speller@univ-pau.fr (A.S.); maitre.julien@univ-pau.fr (J.M.); 2 Department of Biomedical Sciences, University of Padova, 35122 Padova, Italy; alex.rizzato@unipd.it (A.R.); giuseppe.marcolin@unipd.it (G.M.)

**Keywords:** walking downhill, walking uphill, fatigue, postural control, posture, proprioception, vestibular inputs

## Abstract

**Highlights:**

**What are the main findings?**

- Postural balance was disrupted immediately after the completion of the two fatiguing walking sequences, with no difference between walking downhill and walking uphill.
- However, their disruptive factors would differ at the muscular, metabolic and sensory levels.

**What are the implications of the main findings?**

- Walking uphill and walking downhill sequences generated similar muscular and central fatigue.
- Walking uphill could better preserve (or less alter) the vestibular and/or proprioceptive channels contributing to postural balance, compared to walking downhill.

**Abstract:**

Background/Objectives: Walking-induced fatigue disrupts postural balance, but the differentiated effects of walking uphill and downhill remain unclear. The aim was to compare the impact of two walking sequences, either uphill (+10%) or downhill (−20%), with an identical number of steps (7000 steps) on a treadmill at 5.5 km·h^−1^ on postural balance. Methods: Nineteen healthy young participants performed the two walking sequence sessions (56 and 57 min) eight days apart. Eyes-closed bipedal postural balance (in three randomized conditions: an unmanipulated condition, a tendon vibration manipulation condition—TV—and a galvanic vestibular stimulation manipulation condition—GVS), maximal voluntary contraction and central activation ratio were assessed before (PRE), immediately after (POST) and 20 min after (POST20) each walking sequence. Results: Walking uphill and walking downhill sequences generated similar muscular and central fatigue in the POST and POST20 conditions. In the unmanipulated postural condition, postural balance was disrupted after both walking sequences in the POST condition, with no difference between walking downhill and walking uphill. In the manipulated postural conditions, postural balance was modified by the walking sequences. It was disrupted in the presence of GVS in the POST condition with no difference between walking downhill and uphill, whereas it was not disrupted in the presence of TV and was even improved after walking uphill. Conclusions: Although the postural alteration was broadly similar between the two walking sequences, their disruptive factors would differ at the muscular, metabolic and sensory levels.

## 1. Introduction

Fatigue induced by physical exertion disrupts postural balance, even after the most natural physical activities (i.e., requiring no special attention or particular motor skills) such as running and walking [1,2,3,4,5,6,7,8,9,10,11]. However, postural balance is more disrupted after running exercise than after walking exercise [12]. These authors explained this phenomenon by pointing out that the amplitude and acceleration of vertical movements are greater when running than when walking. It was identified that the greater the repetitive vertical movements of the body on the ground, the more the vestibular and proprioceptive sensory functions involved in the regulation of postural balance are impaired [13]. In fact, repetitive vertical accelerations of the head, and especially its abrupt decelerations and the resulting jolts, reduce the sensitivity and/or increase the detection threshold of the otolithic organs and disturb the integration of their signals [1]. Moreover, repetitive vertical movements that generate strong impacts/constraints on the musculoskeletal and articular systems are also likely to degrade proprioception and position sense [14]. The sensitivity of neuromuscular spindles and articular receptors, as well as the accuracy and reliability of their signals and their integration, are impaired [13].

Although walking generally generates less vertical acceleration than running, it is nevertheless likely to disrupt the contribution of vestibular and proprioceptive afferents to the regulation of postural balance [13]. However, depending on whether the walk is uphill or downhill, the sensory contribution associated with fatigue could vary significantly. Walking uphill involves positive mechanical work to shift the centre of gravity, concentric muscle contractions, and short strides that skim the ground, while walking downhill involves negative mechanical work to absorb mechanical energy and control the centre of gravity, eccentric muscle contractions and longer, higher strides [15,16,17,18].

Hence, potential changes in the sensory contribution after walking downhill and walking uphill which induce fatigue could be explained by various factors. The amplitude of repetitive vertical movements and their induced impact on the ground are greater when descending—downhill—than when ascending—uphill—[18,19], which could lead to potentially more significant vestibular disturbance. Moreover, since eccentric muscle actions cause more muscle damage than concentric muscle actions, walking downhill could provoke greater proprioceptive disturbances than walking uphill [16,18,20,21,22,23,24,25]. Eccentric and concentric muscle actions may also have different effects on the level of voluntary activation of the muscles involved. Indeed, while an identical reduction in maximum voluntary contraction (MVC) was observed following eccentric and concentric muscle actions performed separately with the knee extensors, central fatigue was only detected after the eccentric actions [26]. Central fatigue plays a significant role in disrupting the regulation of postural balance [13].

Despite the data mentioned above, it remains unclear whether the effects of walking downhill on postural balance differ from those of walking uphill, since as far as we know, no study has yet directly compared them under conditions of induced fatigue. Therefore, the first aim was to compare the impact of two walking sequences, either uphill or downhill, with an identical number of steps on a treadmill at a constant and identical speed on postural balance in eyes-closed condition, since it is well established that visual information effectively compensates for the decreased effectiveness of other sensory channels [13]. The second aim was to distinguish the effects of the two walking sequences on muscular and central fatigue, as well as on the contribution of vestibular and proprioceptive functions to the regulation of postural balance. The first hypothesis was that walking downhill disrupts postural balance more than walking uphill. The second hypothesis was that walking downhill also disrupts muscular and central fatigue, as well as vestibular and proprioceptive functions, more than walking uphill.

## 2. Method

### 2.1. Participants

Twenty-three healthy young active participants (10 women, 13 men; age: 23 ± 1.5 years; height: 169.35 ± 9.3 cm; weight: 66.07 ± 10.16 kg) were recruited to participate in the study. Exclusion criteria included any history of postural, neurological, musculoskeletal or joint disorders within the 12 months prior to testing. Participants were instructed to avoid intense physical activity for 48 h before each testing session. All participants provided written informed consent prior to participation, in accordance with the Declaration of Helsinki.

### 2.2. Protocol

The experiment aimed to examine potential changes in postural balance and neuromuscular function induced by two walking sequences performed on different slopes (downhill vs. uphill). Eyes-closed bipedal postural balance, isometric maximal voluntary contraction (MVC) and central activation ratio (CAR) were assessed before (PRE), immediately after (POST) and 20 min after (POST20) each walking sequence. Each participant completed both walking sequences in a counterbalanced order, with at least 8 days between sessions. The different stages of the protocol followed the chronological order set out below. On each testing day, participants first underwent a familiarization phase for the postural assessment consisting of two eyes-closed bipedal postural balance trials, followed by baseline postural assessment (PRE). They then performed a 15 min warm-up. Participants then underwent a familiarization phase for the quadriceps MVC and CAR measurements, consisting of progressive quadriceps contractions of increasing intensity up to a maximal effort, followed by MVC and CAR measurements. After completing the walking protocol, MVC and CAR were reassessed, along with postural balance immediately after the walking sequence (POST) and again 20 min later (POST20).

### 2.3. Postural Tests and Sensory Manipulations

For each postural assessment, participants stood barefoot in a bipedal stance, eyes closed, and were instructed to remain as still as possible for 51.2 s on a force platform (PostureWin, Techno Concept, Céreste, France; sampling frequency: 40 Hz; 12-bit A/D conversion), with arms alongside their body. The legs were straight and the feet formed an angle of 30° (inter-malleolar distance of 5 cm). The subjects were similarly placed according to precise marks [27]. Centre of pressure (CoP) displacements of the feet were recorded to compute mean CoP velocity (the total CoP displacement divided by the total period) along the medio-lateral (CoP-ML) and antero-posterior (CoP-AP) axes (mm·s^−1^).

Postural balance was assessed under three randomized conditions: an unmanipulated condition, a tendon vibration manipulation condition (TV manipulation) and a galvanic vestibular stimulation manipulation condition (GVS manipulation). In the unmanipulated condition, no sensory manipulation was triggered. In the TV manipulation condition, bilateral Achilles tendon vibration was applied using inertial vibrators (VB 115, Techno Concept, Mane, France) secured with elastic bands. Vibration frequency was set at 80 Hz [27]. In the GVS manipulation condition, a 3 mA binaural bipolar galvanic vestibular stimulation was delivered using a constant current stimulator (Mio-Ionotens, I-TECH Medical Division, Scorzè, Italy). Polarity was counterbalanced across participants [27].

### 2.4. Warm-Up

As some authors suggest (e.g., ref. [28]), the warm-up followed a certain structure. It consisted of cycling on an ergometer (Monark^®^ Ergomedic E874, Vansbro, Sweden) with progressively increasing intensity over 15 min: 5 min at 50% of heart rate reserve (HRR), 5 min at 70% HRR, 2 min at 80% HRR, followed by 1 min of active recovery and a final 2 min at 80% HRR. Heart rate was continuously monitored using a chest strap (Polar^®^ H10, Kempele, Finland). Participants then performed two sets of 20 full squats without external load, with 1 min of rest between sets. A 10 min seated rest period, without fluid intake, was imposed before the start of the uphill or downhill walking task.

### 2.5. Maximal Voluntary Contraction Assessment

The isometric maximal voluntary contraction (MVC) of the quadriceps was assessed for the dominant leg using a leg extension ergometer (Panatta Sport™, Apiro, Italy) at PRE, POST, and POST20. The device was equipped with one force sensor (Model SSM Series, PM Instrumentation™, Courbevoie, France) attached at the ankle level. Force signals were recorded using a data acquisition system (Biopac MP100, Biopac Systems Inc., Santa Barbara, CA, USA) at a sampling frequency of 200 Hz.

Participants were seated with the hip and knee joints flexed at 90°. The backrest was inclined at 10° (tilted backwards relative to the vertical for the participants), and the seat depth was adjusted to each participant’s thigh length. The trunk and pelvis were stabilized using straps, and the arms were crossed over the chest. After a familiarization period, for the PRE trial, participants performed two maximal 5 s isometric muscular actions, with 30 s of rest between each muscular action. The highest value obtained for each leg was retained for analysis. For the POST and POST20 trials, only one maximal muscular action was performed. Strong verbal encouragement was provided, but no performance feedback was given.

### 2.6. Central Activation Ratio Assessment

Immediately after MVC assessment, central activation ratio (CAR) was evaluated on the dominant leg at PRE, POST and POST20. To quantify central activation failure, a supramaximal electrical stimulation (ES) was manually delivered once force had reached a plateau (approximately 3 s after contraction onset) and maintained for 2 s. CAR was calculated as follows [29]:

CAR = MVC/(MVC + ES)

MVC represents the voluntary force and MVC + ES the combined voluntary and electrically evoked force. When no additional force was elicited during stimulation, CAR was equal to 1.0, indicating complete voluntary activation. Electrical stimulation was delivered using a high-voltage constant-current stimulator (DS7, Digitimer, Hertfordshire, UK). Two rectangular adhesive electrodes (Dura-Stick^®^ Plus, 5 × 10 cm, Cefar-Compex, Vista, CA, USA) were positioned over the motor points of the vastus medialis and rectus femoris muscles. Stimulation consisted of a biphasic symmetrical rectangular waveform (pulse duration: 450 μs, frequency: 80 Hz, intensity: 80 mA).

### 2.7. Walking Sequences

Participants completed two walking sequences of 7000 steps on a force-distribution treadmill (FDM-T, Zebris Medical, Isny im Allgäu, Germany). The sequences were performed at a constant speed of 5.5 km·h^−1^, under two slope conditions: downhill (−20%) and uphill (+10%). The number of steps was monitored using a pedometer (Onwalk One Plus, Decathlon, Villeneuve-d’Ascq, France). To control for potential confounding effects of footwear, all participants wore standardized shoes (Aquashoes 100, SUBEA, Decathlon, Villeneuve-d’Ascq, France).

### 2.8. Data Processing

To characterize exercise effect and recovery, signed changes (Δ) were calculated for each parameter within each condition, namely unmanipulated, TV and GVS, as the difference between time points (POST-PRE and POST20-POST). Two transitions were considered: PRE to POST (Δ PRE → POST) and POST to POST20 (Δ POST → POST20), reflecting exercise effect and recovery phase, respectively.

To quantify the specific effects of sensory manipulations on postural balance, TV responses and GVS responses at each time point (PRE, POST and POST20) were calculated relative to the unmanipulated condition recorded before exercise (Unmanipulated PRE). Their signed changes, Δ PRE → POST and POST → POST20, were also calculated. When referenced to the unmanipulated condition recorded before exercise, TV and GVS responses specifically quantified the postural responses, independently of the baseline level, induced by tendon vibration and galvanic vestibular stimulation.

For each participant and slope condition, GVS and TV responses were computed as follows:

TV response (PRE, POST or POST20) = TV condition − Unmanipulated PRE

GVS response (PRE, POST or POST20) = GVS condition − Unmanipulated PRE

### 2.9. Statistical Analysis

Statistical analyses were performed using R statistical software (v 4.5.2). The significance level was set at α < 0.05. The experimental design included Time (PRE, POST, POST20) and Slope (uphill vs. downhill) as within-subject factors, as well as their interaction. Given the repeated-measures design and the non-normal distribution of the data, a rank-based nonparametric repeated-measures ANOVA was performed using the nparLD package (v 2.2) for R [30] to test the main effects of time and slope and their interactions. Test statistics are reported as ANOVA-type statistics (ATS), as provided by the nparLD package. For the neuromuscular parameters, including MVC and the CAR, analyses were performed separately for each variable. For the postural balance parameters (CoP-ML and CoP-AP velocities), analyses were conducted separately for each condition (unmanipulated, TV and GVS) and for their computed responses (TV response and GVS response).

When a significant Time × Slope interaction was observed, planned post hoc comparisons were conducted using paired Wilcoxon signed-rank tests to examine temporal changes within each slope condition (PRE vs. POST, POST vs. POST20, and PRE vs. POST20), as well as differences between slopes at each time point. These comparisons were adjusted using the Holm–Bonferroni correction [31].

When the interaction effect was not statistically significant but a main effect of time was identified, additional planned comparisons between time points were performed to describe the overall temporal evolution, irrespective of slope.

In addition, signed changes (Δ), for the neuromuscular and the postural balance parameters, were compared between uphill and downhill conditions using paired Wilcoxon signed-rank tests with Holm–Bonferroni correction [31].

As HR data were normally distributed, heart rate values recorded at 3500 and 7000 steps were compared between uphill and downhill conditions using *T*-tests with Holm–Bonferroni correction [31].

## 3. Results

Only 19 of the 23 participants entirely completed the protocol. Four participants were unable to finish the walking uphill sequences. Consequently, data from nineteen participants (9 women, 10 men; age: 23 ± 1.5 years; height: 170.35 ± 9.5 cm; weight: 66.09 ± 9.8 kg) were included in the final analyses.

### 3.1. Walking Durations and Heart Rate

The heart rate (HR) at rest of the participants was 56 ± 6 bpm. Their HRs were significantly different at 3500 steps, 165 ± 15 bpm for uphill vs. 107 ± 10 bpm for downhill [t(16) = 17.01, *p* < 0.001] and at 7000 steps, 170 ± 14 bpm for uphill vs. 109 ± 10 for downhill [t(16) = 20.12, *p* < 0.001]. The duration of the walking sequences was 56 ± 0.0024 min for the uphill and 57 ± 0.0024 min for the downhill.

### 3.2. Maximal Voluntary Contraction

**Figure 1:**
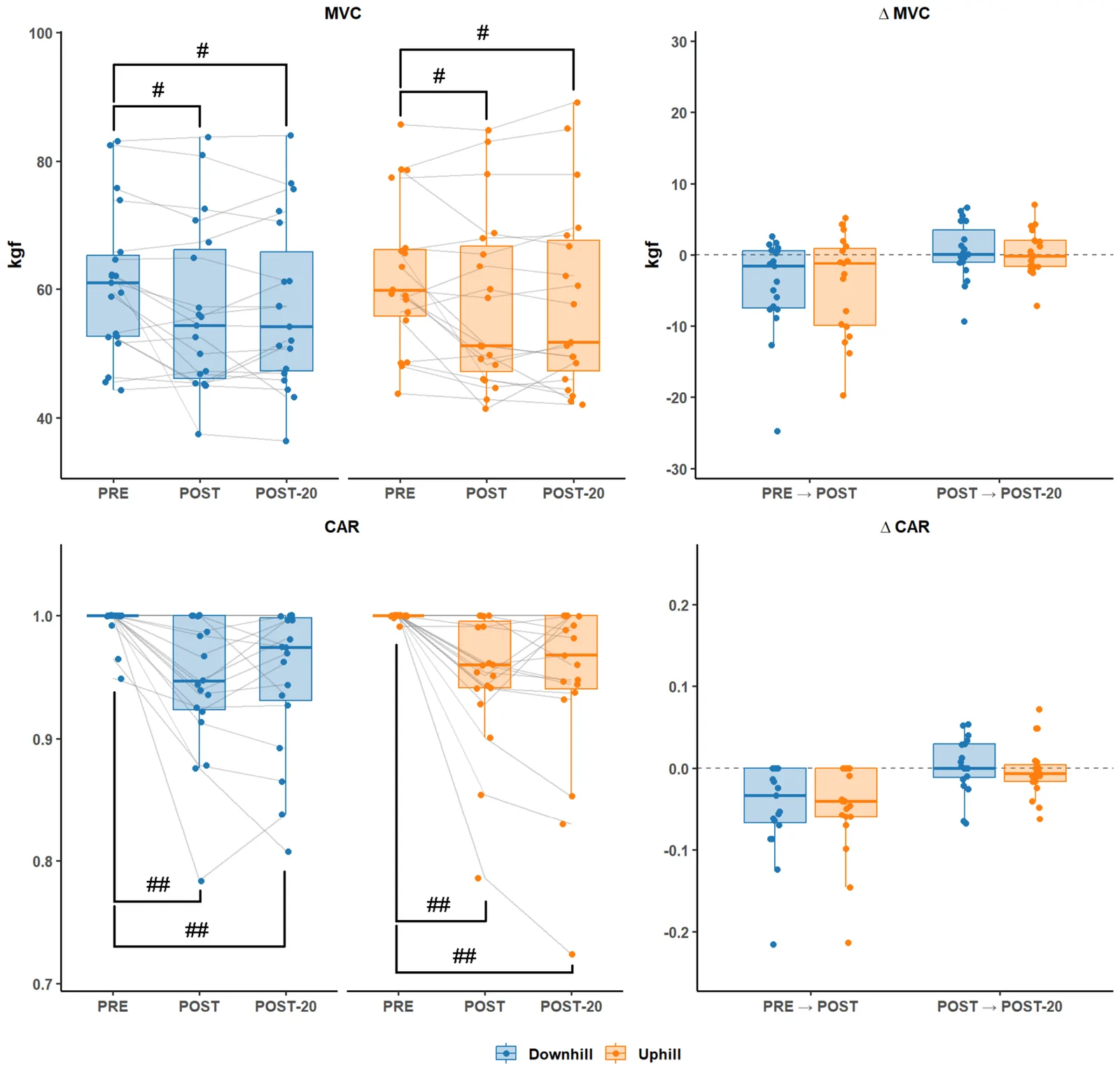
Isometric maximal voluntary contraction (MVC) and central activation ratio (CAR) of the quadriceps during uphill and downhill conditions at PRE, POST and POST20, with signed changes (Δ) evolution between time points. n = 19. #, ## indicate significant post hoc overall temporal evolution, irrespective of slope, *p* < 0.05 and *p* < 0.01, respectively.

For the dominant limb (Figure 1), a significant main effect of Time [ATS (1.31) = 6.70, *p* = 0.005] was observed, indicating a similar temporal evolution irrespective of slope. Post hoc analyses revealed significant decreases between PRE and POST and between PRE and POST20.

For the non-dominant limb, only a significant difference was revealed between the uphill and downhill protocol concerning the Δ PRE → POST.

### 3.3. Central Activation Ratio

A significant main effect of Time [ATS(1.65) = 34.54, *p* < 0.001] was observed for the central activation ratio (Figure 1), indicating a similar temporal evolution irrespective of slope. Post hoc analyses revealed significant decreases between PRE and POST and between PRE and POST20.

### 3.4. CoP in Unmanipulated Condition

**Figure 2:**
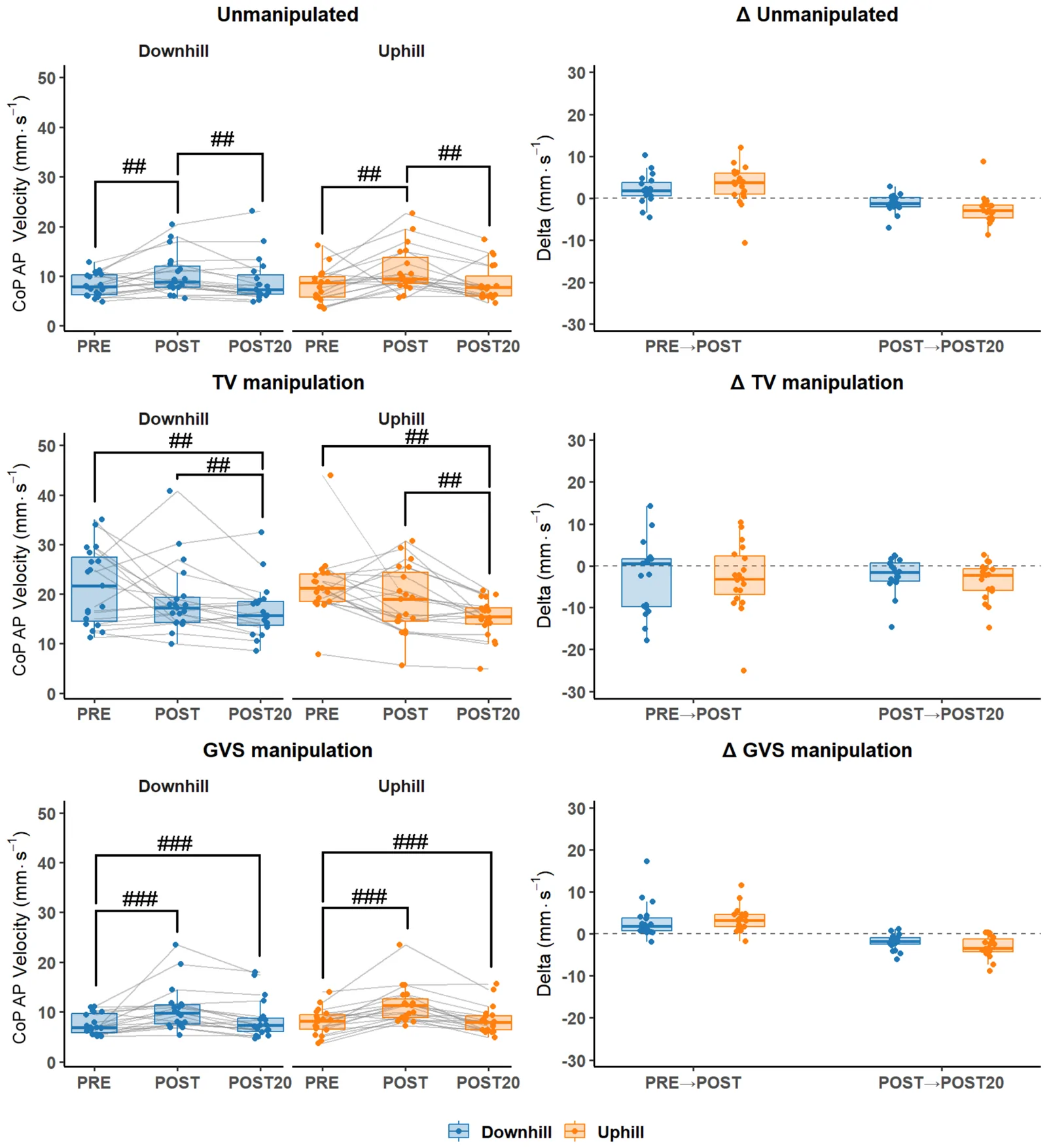
CoP-AP velocity during uphill and downhill conditions under unmanipulated, tendon vibration (TV) and galvanic vestibular stimulation (GVS) conditions at PRE, POST and POST20, with signed changes (Δ). n = 19. ##, ### indicate significant post hoc overall temporal evolution, irrespective of slope, *p* < 0.01 and *p* < 0.001, respectively.

The CoP-AP velocity (Figure 2) presented a significant main effect of time [ATS(1.46) = 8.34, *p* = 0.001], indicating a similar temporal evolution irrespective of slope. Post hoc analyses showed significant differences between PRE and POST and between POST and POST20.

### 3.5. COP in Tendon Vibration Manipulation Condition

**Figure 3:**
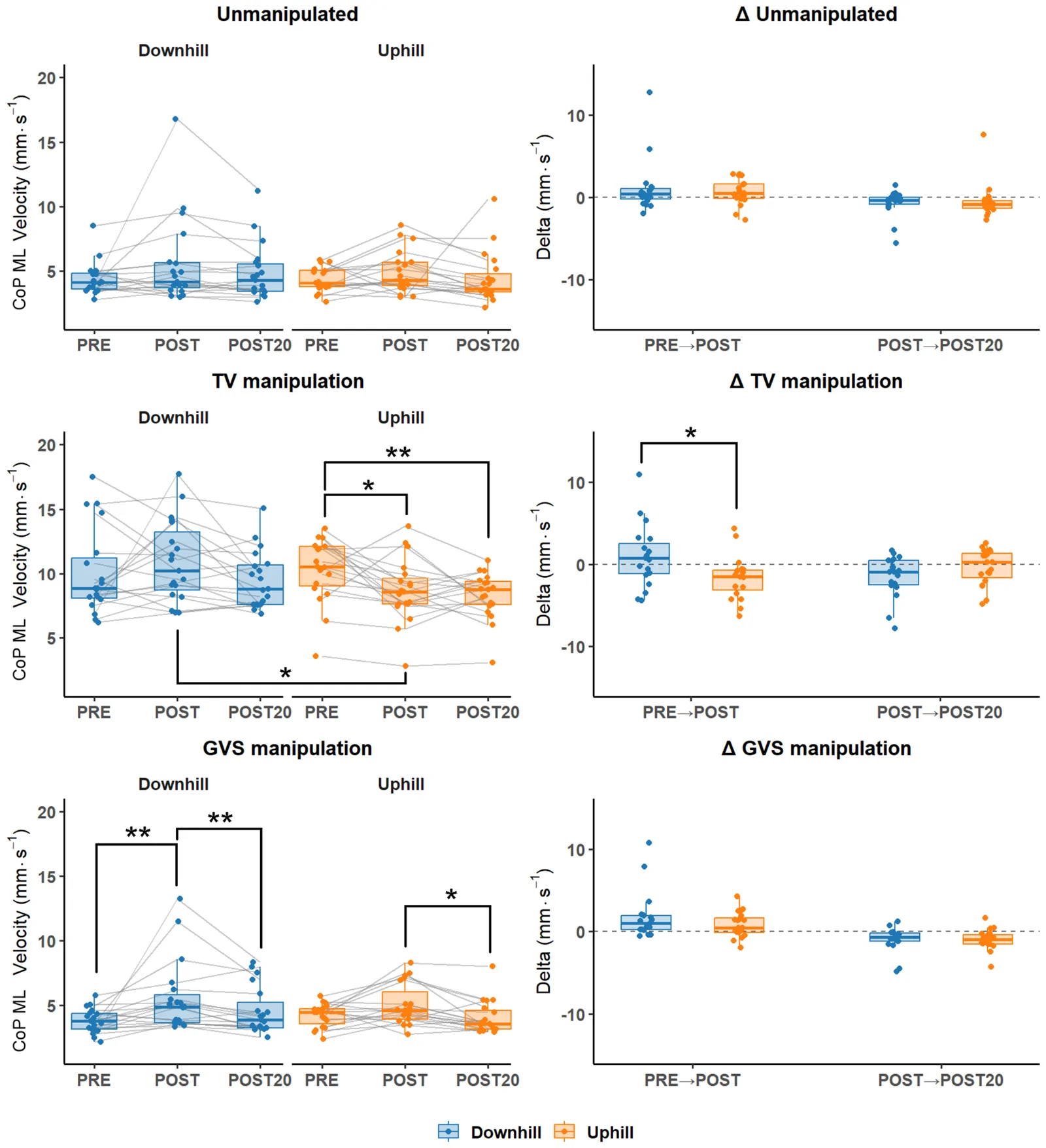
CoP-ML velocity during uphill and downhill conditions under unmanipulated, tendon vibration (TV) and galvanic vestibular stimulation (GVS) conditions at PRE, POST and POST20, with signed changes and TV responses. n = 19. * and ** indicate significant post hoc comparisons (*p* < 0.05 and *p* < 0.01, respectively).

The CoP-ML velocity (Figure 3) presented a significant Time × Slope interaction [ATS(1.63) = 9.38, *p* < 0.001] indicating that the temporal evolution differed between uphill and downhill conditions. Post hoc analyses indicated significant differences across time within the uphill condition between PRE and POST and between PRE and POST20. Comparisons between slopes revealed significant differences at POST. In addition, analysis of signed change, Δ TV manipulation (Figure 3), revealed significant differences between slope conditions, during the phase PRE → POST, for CoP-ML velocity.

For CoP-AP velocity (Figure 2), a significant main effect of time was observed [ATS(1.37) = 16.30, *p* < 0.001], indicating a similar temporal evolution irrespective of slope. Post hoc analyses showed significant differences between POST and POST20 and between PRE and POST20.

**Figure 4:**
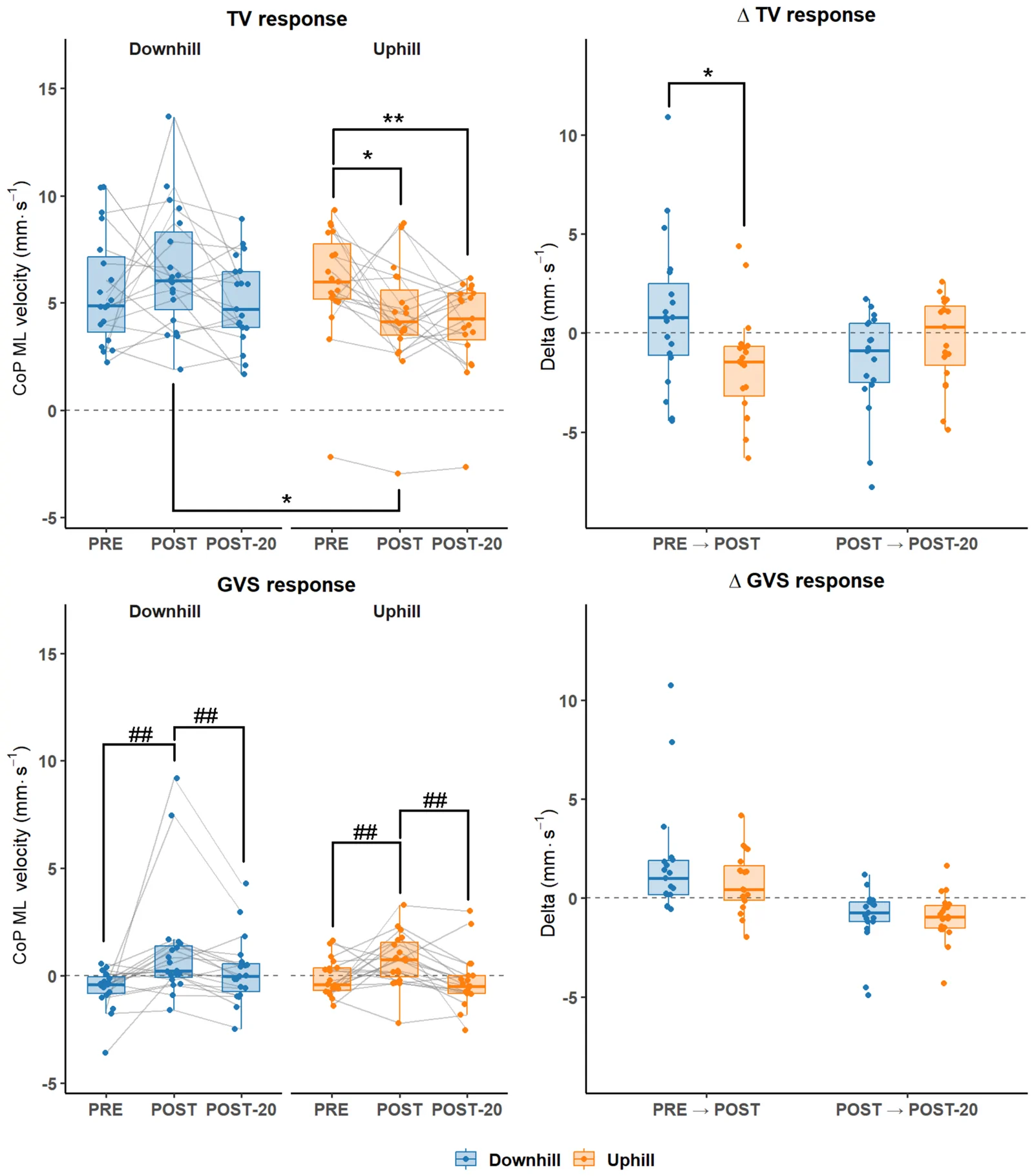
CoP-ML velocity during uphill and downhill conditions for the tendon vibration (TV) and galvanic vestibular stimulation (GVS) responses at PRE, POST and POST20, with signed changes (Δ). n = 19. * and ** indicate significant post hoc comparisons (*p* < 0.05 and *p* < 0.01, respectively) ## indicate significant post hoc overall temporal evolution, irrespective of slope, *p* < 0.01, respectively.

**Figure 5:**
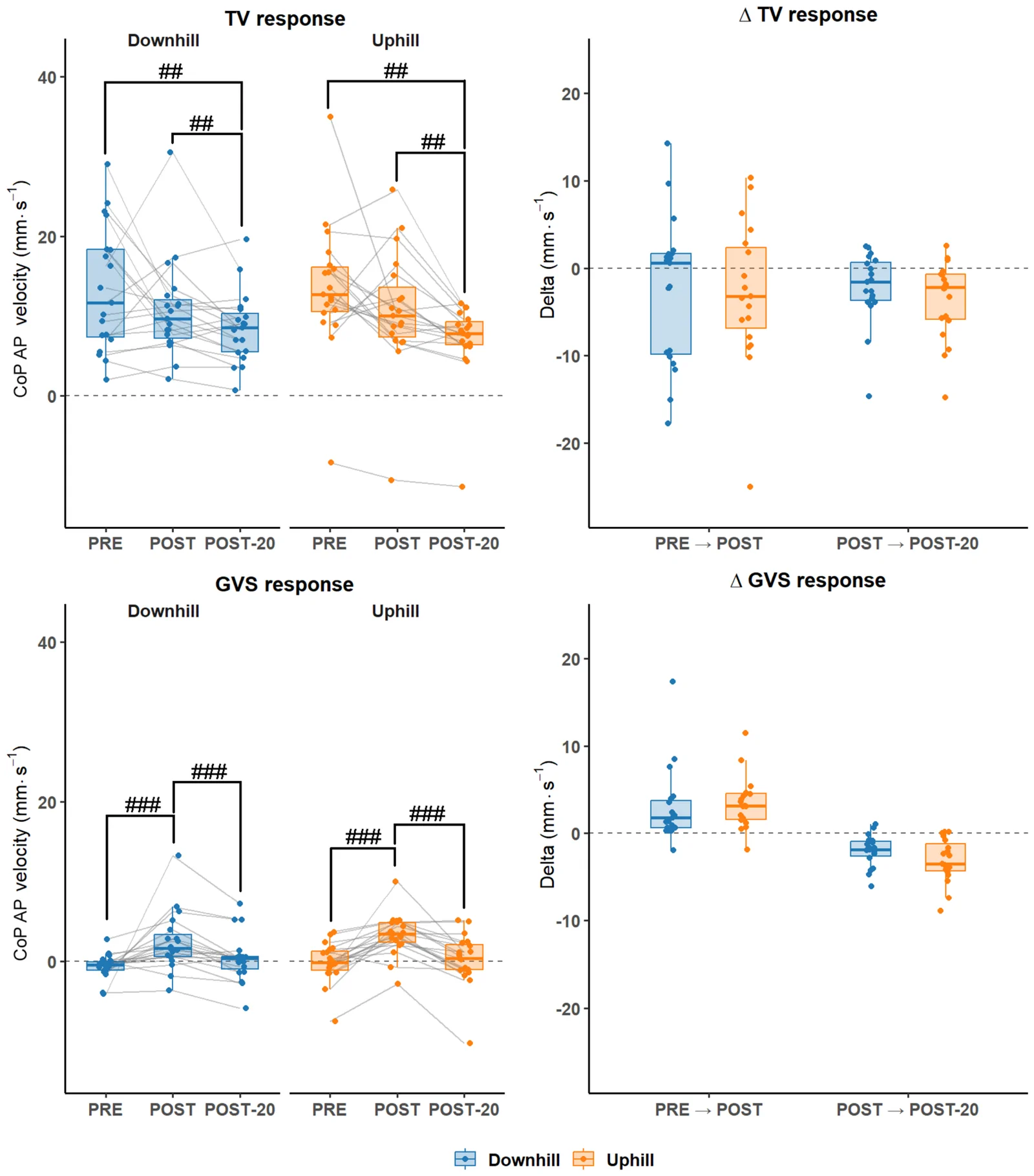
CoP-AP velocity during uphill and downhill conditions for the tendon vibration (TV) and galvanic vestibular stimulation (GVS) responses at PRE, POST and POST20, with signed changes (Δ). n = 19. ##, ### indicate significant post hoc overall temporal evolution, irrespective of slope, *p* < 0.01 and *p* < 0.001, respectively.

Concerning the TV response, CoP-ML velocity (Figure 4) presented a significant Time × Slope interaction [ATS(1.52) = 7.94, *p* < 0.001], indicating that the temporal evolution differed between uphill and downhill conditions. Post hoc analyses indicated significant differences across time within the uphill condition between PRE and POST and between PRE and POST20. Comparisons between slopes revealed significant differences at POST. In addition, the analysis of signed change, Δ TV response (Figure 4), revealed significant differences between slope conditions, during the phase PRE → POST, for CoP-ML velocity. Furthermore, for CoP-AP velocity (Figure 5), a significant main effect of time was observed [ATS(1.35) = 16.13, *p* < 0.001], indicating a similar temporal evolution irrespective of slope. Post hoc analyses showed significant differences between POST and POST20 and between PRE and POST20.

### 3.6. CoP in Galvanic Vestibular Stimulation Condition

The CoP-ML velocity (Figure 3) presented a significant Time × Slope interaction [ATS(1.81) = 3.12, *p* = 0.049], indicating that the temporal evolution differed between uphill and downhill conditions. Post hoc analyses indicated significant differences across time within the uphill condition between POST and POST20 and within the downhill condition between PRE and POST and between PRE and POST20. A significant main effect of time was also observed [ATS(1.67) = 7.92, *p* < 0.001].

For CoP-AP velocity (Figure 2), a significant main effect of time was observed [ATS(1.82) = 18.68, *p* < 0.001], indicating a similar temporal evolution irrespective of slope. Post hoc analyses showed significant differences between PRE and POST and between PRE and POST20.

Concerning the GVS response, CoP-ML and CoP-AP velocities (Figure 4 and Figure 5) presented a significant main effect of time [ATS(1.55) = 11.28, *p* < 0.001 and ATS(1.54) = 15.72, *p* < 0.001, respectively], indicating a similar temporal evolution irrespective of slope. Post hoc analyses showed significant differences between PRE and POST and between POST and POST20 for the CoP-ML and CoP-AP velocities.

## 4. Discussion

The first aim was to compare the impact of two walking sequences, either uphill or downhill, with an identical number of steps on a treadmill at a constant and identical speed on postural balance in eyes-closed condition. The second aim was to distinguish the effects of the two walking sequences on muscular and central fatigue, as well as on vestibular and proprioceptive contributions in the postural regulation. As expected, the results show that both walking sequences (uphill and downhill) induced fatigue, as evidenced by reductions in MVC and CAR under the POST and POST 20 conditions. However, these reductions were similar for both walking downhill and uphill. In a state of proven fatigue and in the unmanipulated postural condition (without sensory manipulation), postural balance was disrupted in the POST condition after both walking sequences in the sagittal plane, with no difference between walking downhill and walking uphill. In the manipulated postural conditions (with sensory manipulation), the results showed that with GVS, postural balance was disrupted in the POST condition after both walking sequences on the frontal and sagittal planes, whereas it was not disrupted with TV and was even significantly improved after walking uphill on the frontal plane in comparison with walking downhill. Overall, neither of the two proposed hypotheses was supported.

The results indicate that the MVC decreased after both walking sequences. This means that walking uphill and downhill reduced the muscle strength of the knee extensors and thus induced a state of fatigue (i.e., reduction in number of motor units recruited and/or discharge action potentials) in the participants [32]. Previous studies have already revealed that walking downhill (−10%) and/or walking level (0%) decreased MVC in healthy older adults and adolescents with spastic cerebral palsy for shorter durations and lower walking speeds, i.e., 30 min and 15 min at a comfortable/acceptable speed versus 56 min at 5.5 km·h^−1^ in the present study [8,33]. Moreover, the reduction in the CAR indicates that the participants exhibited central fatigue. That is generally attributed to alterations in the activation of the primary motor cortex, propagation of the descending command and/or excitability of spinal motoneurons [34,35]. Central fatigue is known to impair the ability to finely regulate postural balance [13]. Although the walking sequences induced muscular and central fatigue for both types of walking, no difference was observed between walking downhill and walking uphill. These findings suggest that the two types of walking would not have a different impact on the motor output of the postural function.

In the unmanipulated postural condition, the CoP-AP velocity increased in the POST condition for both types of walking. This means that postural balance was disrupted in the sagittal plane immediately after the completion of walking sequences. This result is consistent with previous studies that have examined the effects of walking effort on postural balance in healthy and pathological subjects [5,6,8,9,33,36]. In return, the CoP-ML velocity did not increase in the POST condition for both types of walking. This suggests that the effects induced by the two walking sequences on postural balance are weak or restricted to the sagittal plane. If these effects are weak, the static postural condition may have been too simple to discriminate the difference between the PRE condition (baseline postural assessment) and the POST condition; only a dynamic postural condition and/or a non-linear analysis technique of the displacement of the CoP may be more sensitive for detecting the differences between the conditions before and after a walking exercise [6,37]. If these effects only appear on the sagittal plane, this could be because walking relies primarily on the activity of the extensor muscles of the hip, knee and ankle, which act mainly on the sagittal plane [15]. Hence, the fatigue of these extensor muscles would mainly disrupt postural balance on the sagittal plane.

In the POST 20 condition, the CoP-AP velocity is brought back to its base value; more precisely, it is no longer significantly different from the PRE condition. This could be explained by the fact that weak or subtle effects induced by fatigue would only last for a short time. Indeed, exercises inducing only small reductions in force production, on the order of 7% of the MVC as observed in the present study, generally have no effect on postural balance, or, when an effect is observed, it lasts only a few minutes rather than as long as 20 min, except following very long exercises [13]. In older participants, Walsh et al. [6] however reported that 15 min of recovery was insufficient to return to baseline after an alteration of postural balance induced by 30 min of walking uphill and level. In the healthy young participants like those of this study, this rapid recovery is not surprising, since their postural function is able to quickly compensate for the disruptive effects induced by walking exercise and to adequately regulate postural balance [9].

The absence of difference between the effects induced by walking downhill and those induced by walking uphill means that walking downhill did not disturb postural balance more than walking uphill, despite greater impacts on the ground and more pronounced eccentric actions (i.e., stronger mechanical constraints/stresses) of the propulsive muscles involved not only in walking but also in postural regulation. However, Hill et al. [8] reported that the effects of muscle damage induced by eccentric actions on postural balance would only appear 24 h after walking exercise (30 min) and would persist for at least 48 h. Since postural balance was assessed only immediately after both types of walking and after 20 min of recovery, the potential/possible delayed effects of eccentric muscle actions could not be observed in the present study. Moreover, walking uphill involved positive mechanical work to shift the centre of gravity while walking downhill involved negative mechanical work to absorb mechanical energy and control the centre of gravity [15,16,17,18]. This means that the effort required to complete the walking sequences and the induced metabolic load were greater during the walking uphill sequence than during the downhill walking sequence, as evidenced by the significant differences between the HR values in the middle (3500 steps) and at the end (7000 steps) of the walking sequences. In addition, four participants were unable to complete the walking uphill sequence. Given that a significant metabolic load causes postural balance disruption due to the large cardiorespiratory responses and fluid movements to exercise and their effects on postural sway [13], the disruptive effects induced by the walking uphill sequence on postural balance could be of a magnitude similar to those induced by the walking downhill sequence with greater mechanical stresses, particularly in the POST condition.

Moreover, the comparison of the contribution of vestibular and proprioceptive afferents to the regulation of postural balance after fatigue induced by walking downhill and walking uphill was studied using sensory manipulations. The objective through the manipulation of sensory inputs is to highlight the importance (or predominance) of the contribution of the manipulated sensory channel and/or the capacity of the unmanipulated sensory channel(s) to compensate for the disruptive effects of the manipulated sensory channel in postural regulation [27]. Knowing that visual information effectively compensates for the decreased effectiveness of other sensory channels in cases of fatigue [13], in the closed-eye postural condition (no visual inputs) this compensation phenomenon could not occur. Hence, GVS highlights the importance of vestibular inputs and/or the increased contribution of proprioceptive inputs while TV highlights the importance of proprioceptive inputs and/or the increased contribution of vestibular inputs in postural regulation [27]. Overall, the results show that postural balance was disrupted in the presence of GVS in the POST condition after both walking sequences on the frontal and sagittal planes, whereas it was not disrupted in the presence of TV and was even improved after walking uphill on the frontal plane. When referenced to the unmanipulated condition recorded before exercise, TV and GVS responses specifically quantified the postural responses to the sensory manipulations. The results indicate that the GVS response across the three conditions (PRE, POST and POST 20) showed no significant differences between the two walking sequences, indicating that GVS manipulation did not accentuate the fatigue-related alterations in postural balance. The shock-absorbing characteristics of the treadmill used in this study, which differ from firmer surfaces typically encountered under ecological conditions, could have attenuated the mechanical impacts, thereby limiting vestibular disturbances. In return, the TV response did not reveal any disturbance in postural balance immediately after both walking sequences and even showed improved postural performance after uphill walking compared with walking downhill in the frontal plane. This finding indicates that fatigue induced by walking uphill and downhill differentially affects postural regulation under proprioceptive sensory manipulation. The fact that postural balance was improved after walking uphill on the frontal plane in the presence of TV would mean that the disruptive effects of the proprioceptive channel could be overcompensated by an increased contribution of vestibular inputs and/or an reweighting of proprioceptive inputs from myoarticular receptors located above the ankle. Walking uphill could better preserve (or less alter) the vestibular and/or proprioceptive channels contributing to postural balance, compared to walking downhill.

## 5. Study Limitations

One might wonder why the slope percentages were not symmetrical (similar) between the two walking sequences (e.g., +20% versus −20%). In fact, a +20% slope would have been too demanding, since 4 of the 23 participants were already unable to complete the uphill walking sequences at +10%. Conversely, a −10% slope would not have been sufficiently challenging (disruptive) for the downhill walking sequence. Moreover, it is likely that, under ecological conditions, the observed effects would have been more pronounced in both walking sequences. Indeed, the shock-absorbing characteristics of the treadmill used in this study, which differ from the firmer surfaces typically encountered under ecological conditions, may have reduced the mechanical impacts associated with the two walking sequences, thereby limiting their disruptive effects on the postural function. However, such conditions would have been considerably more difficult to standardize and control in practice.

Moreover, the effects of the two walking conditions were measured only immediately after their completion and after 20 min of recovery. Additional measurements taken 6, 12, 24 and 48 h after the completion of the two walking trials would have probably provided more conclusive results. Furthermore, although visual information compensates for the decreased effectiveness of other sensory channels in case of fatigue, it cannot be ruled out that postural measurements taken with eyes open would have given different results. At last, the present study was conducted exclusively with healthy, young, physically active participants. It is therefore possible that different results would have been obtained in other populations, such as older adults, frail individuals, or pathological participants. The results obtained with this protocol should not be generalized to the general population.

## 6. Conclusions

Postural balance was disrupted immediately after the completion of the two fatiguing walking sequences, with no difference between walking downhill and walking uphill. Although the postural alteration was broadly similar between the two walking sequences, their disruptive factors would differ at the muscular, metabolic and sensory levels. Further studies will be needed to precisely quantify the differentiated effects on each of these factors.

## Data Availability

The data presented in this study are available from the corresponding author upon reasonable request. The data are not publicly available due to privacy or ethical restrictions.
